# Glutathione S-transferase pi 1 variant and squamous cell carcinoma susceptibility: a meta-analysis of 52 case-control studies

**DOI:** 10.1186/s12881-019-0750-x

**Published:** 2019-01-21

**Authors:** Shuang Wang, Jingqi Zhang, Fan Jun, Zhijie Bai

**Affiliations:** 1 Department of Plastic and Burn Surgery, Tianjin First Center Hospital, Tianjin, 300192 China; 2 Department of Urology Surgery, Tianjin First Center Hospital, Tianjin, 300192 China

**Keywords:** *GSTP1*, Polymorphism, Squamous cell carcinoma, Susceptibility

## Abstract

**Background:**

There are several meta-analyses on the genetic relationship between the rs1695 polymorphism within the *GSTP1* (glutathione S-transferase pi 1) gene and the risk of different SCC (squamous cell carcinoma) diseases, such as ESCC (oesophageal SCC), HNSCC (head and neck SCC), LSCC (lung SCC), and SSCC (skin SCC). Nevertheless, no unified conclusions have been drawn.

**Methods:**

Herein, an updated meta-analysis was performed to evaluate the probable impact of *GSTP1* rs1695 on the susceptibility to different SCC diseases under six genetic models (allele, carrier, homozygote, heterozygote, dominant, and recessive). Three online databases, namely, PubMed, WOS (Web of Science), and Embase (Excerpta Medica Database), were searched.

**Results:**

Initially, we obtained a total of 497 articles. Based on our selection criteria, we eventually included 52 case-control studies (9763 cases/15,028 controls) from 47 eligible articles. As shown in the pooling analysis, there was no difference in the risk of overall SCC disease between cases and controls [allele, *P*_a_ (*P* value of association test) = 0.601; carrier, *P*_a_ = 0.587; homozygote, *P*_a_ = 0.689; heterozygote, *P*_a_ = 0.167; dominant, *P*_a_ = 0.289; dominant, *P*_a_ = 0.548]. Similar results were obtained after stratification by race (Asian/Caucasian), genotyping, control source, and disease type (ESCC/HNSCC/LSCC/SSCC) (all *P*_a_ > 0.05).

**Conclusion:**

The rs1695 polymorphism within the *GSTP1* gene is not associated with the risk of overall SCC or a specific SCC type, including ESCC, HNSCC, LSCC, and SSCC.

## Background

SCC (squamous cell carcinoma), also termed “epidermal carcinoma,” is a malignant tumour that takes part in epidermis or adnexal cells and exhibits distinct degrees of keratosis [1–3]. SCC exists in the squamous epithelium of several places, e.g., skin, mouth, lung, lips, oesophagus, cervix, and vagina [4–6]. Based on GWAS (genome-wide association study) data, more and more reported genetic polymorphisms are believed to contribute to the aetiologies of different SCC types. For instance, a series of genes, including *CADM*1 (cell adhesion molecule 1), *AHR* (aryl hydrocarbon receptor), and *SEC16A* (SEC16 homolog A, endoplasmic reticulum export factor), may be related with the risk of SCC [7]. Two variants within the *KLF5* (Kruppel-like factor 5) gene on chromosome 13q22.1, namely, rs1924966 and rs115797771, may be relevant to ESCC (oesophageal SCC) susceptibility [8]. Herein, we determined whether *GSTP1* (glutathione S-transferase pi 1) gene polymorphism is associated with the susceptibility to different SCC patterns.

*GSTP1*, a member of the GST (glutathione S-transferase) family in humans, is associated with the biological detoxification or biotransformation process through catalysing the conjugation of many hydrophobic and electrophilic compounds with reduced glutathione [9, 10]. The *GSTP1* gene, which is located on human chromosome 11q13, comprises seven exons and six introns [11]. Two common polymorphisms, namely, rs1695 A/G polymorphism in exon five (p.Ile105Val) and rs1138272 C/T polymorphism in exon six (p.Ala114Val), have been reported [12, 13].

Several SCC/*GSTP1* rs1695-associated meta-analyses with conflicting conclusions have been reported. For instance, in 2009, Zendehdel et al. enrolled three case-control studies [14–16], performed a meta-analysis to assess the association between *GSTP1* rs1695 and ESCC risk in Caucasian populations, and found a borderline significant association [16]. In 2014, Song et al. enrolled 21 case-control studies to perform a meta-analysis concerning the role of the *GSTP1* rs1695 polymorphism in the risk of oesophageal cancers, including EAC (oesophageal adenocarcinoma) and ESCC [17]. The subgroup meta-analysis of ESCC containing thirteen case-control studies showed a positive correlation, particularly in the Caucasian population [17]. However, in 2015, Tan et al. performed another meta-analysis with twenty case-control studies on overall oesophageal cancer and reported negative results in both ESCC and EAC subgroups [18]. Accordingly, we performed an updated meta-analysis with a relatively larger sample size to reevaluate the potential impact of the *GSTP1* rs1695 A/G polymorphism on the susceptibility to SCC diseases, mainly including ESCC, SSCC, HNSCC (head and neck SCC), and LSCC (lung SCC).

## Methods

### Electronic database retrieval

We reviewed three on-line databases, including PubMed, WOS (Web of Science), and Embase (Excerpta Medica Database), through January 2018 using the following main search keywords: Carcinoma, Squamous Cell; Carcinomas, Squamous Cell; Squamous Cell Carcinomas; Squamous Cell Carcinoma; Carcinoma, Squamous; Carcinomas, Squamous; Squamous Carcinoma; Squamous Carcinomas; Carcinoma, Epidermoid; Carcinomas, Epidermoid; Epidermoid Carcinoma; Epidermoid Carcinomas; Carcinoma, Planocellular; Carcinomas, Planocellular; Planocellular Carcinoma; Planocellular Carcinomas; SCC; GSTP1; Glutathione S-Transferase pi; Glutathione S Transferase pi; GST Class-phi; Class-phi, GST; GST Class phi; Glutathione Transferase P1–1; Glutathione Transferase P1 1; Transferase P1–1, Glutathione; GSTP1 Glutathione D-Transferase; D-Transferase, GSTP1 Glutathione; GSTP1 Glutathione D Transferase; Glutathione D-Transferase, GSTP1; Polymorphism; Polymorphism, Genetic; Polymorphisms, Genetic; Genetic Polymorphisms; Genetic Polymorphism; Polymorphism (Genetics); Polymorphisms (Genetics); and Polymorphism; Polymorphisms.

### Eligible article screening

We performed a literature search and screened the retrieved articles as per the PRISMA (preferred reporting items for systematic reviews and meta-analyses) guidelines [19]. Selection criteria included duplicated articles; data from animal or cell experiments; meeting abstract or meta-analysis; review, trials or case reports; data of GSTP1 expression; not SCC or GSTP1; lack confirmed histopathological data; combined GA + AA genotype frequency; without the control data; and *P* value of HWE (Hardy-Weinberg equilibrium) less than 0.05. Eligible case-control studies provided sufficient genotype frequency data of the *GSTP1* gene rs1695 polymorphism in each case and control group.

### Data extraction

Two investigators independently extracted the data and evaluated the methodological quality of each article by means of the NOS (Newcastle-Ottawa Scale) system. One table contains the following basic information: first author, publication year, region, race, genotyping assay, genotype frequency, disease type, control source, *P* values of HWE, study number, and sample size of the case/control.

### Data synthesis

We utilized STATA software (StataCorp LP, College Station, TX, USA) for the following statistical analyses. The allele (allele G vs. A), carrier (carrier G vs. A), homozygote (GG vs. AA), heterozygote (AG vs. AA), dominant (AG + GG vs. AA), and recessive (GG vs. AA+AG) models were utilized to target the *GSTP1* gene rs1695 G/A polymorphism. We calculated the OR (odds ratio), 95% CIs (confidence intervals) and *P*_a_ (*P* value of association test) values to estimate the association. When the *P*_h_ (*P* value of heterogeneity) was > 0.1 or I^2^ was < 50.0%, a fixed-effects model was adopted. Otherwise, a random-effects model was selected.

Considering the factors of race, genotyping assay, control source, and disease type, we performed the corresponding subgroup meta-analyses. We also carried out Egger’s/Begg’s tests to determine a potential publication bias. The presence of a publication bias was considered when *P*_E_ (*P* value of Egger’s test) and *P*_B_ (*P* value of Begg’s test) were below 0.05. Sensitivity analysis was applied to assess data stability and robustness.

## Results

### Article retrieval and screening

**Fig. 1:**
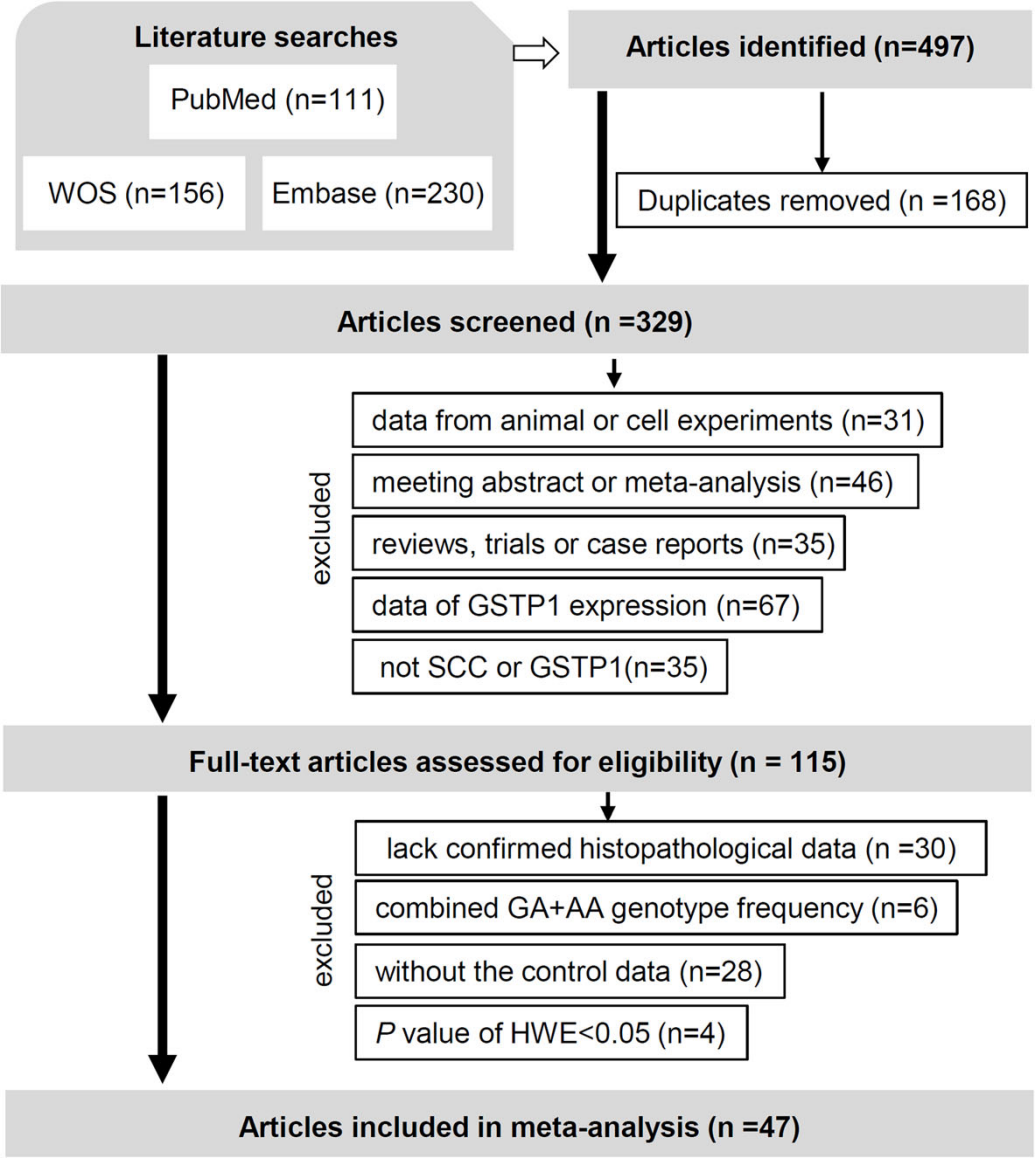
Flow chart of eligible article selection

The article retrieval and selection processes during our meta-analysis were conducted as described in the flow chart shown in Fig. 1. After our literature search, a total of 497 articles were obtained. Then, 168 articles with duplicated data and 214 articles meeting the exclusion criteria were excluded. Next, we assessed the eligibility of the remaining 115 full-text articles. After the exclusion of 68 ineligible articles, a total of 47 articles containing 52 case-control studies [14–16, 20–63] were ultimately recruited for our meta-analysis. Table 1 summarizes the extracted basic information.

**Table 1:** Basic information of the eligible articles in the meta-analysis

First author	Year	Region	Race	Assay	Case			Disease type	Control			Control source	*P*_HWE_
					AA	AG	GG		AA	AG	GG		
Abbas	2004	France	Caucasian	PCR-RFLP	21	21	3	ESCC	59	56	9	PB	0.38
Cabelguenne	2001	France	Caucasian	PCR-RFLP	89	57	16	HNSCC	146	139	25	HB	0.31
Cai	2006	China	Asian	PCR-RFLP	143	58	3	ESCC	265	116	12	PB	0.87
Cho	2006	Korea	Asian	Gene sequencing	201	85	7	HNSCC	211	112	10	HB	0.29
Dura	2013	Netherlands	Caucasian	PCR	48	42	15	ESCC	246	261	84	PB	0.27
Dzian	2012	Netherlands	Caucasian	PCR-RFLP	56	45	11	LSCC	153	115	22	PB/HB	0.95
Evans	2004	USA	Caucasian	PCR-RFLP	123	132	27	HNSCC	97	85	24	PB	0.42
Fryer	2005	Australia	Caucasian	PCR-RFLP	59	51	18	SSCC	95	90	25	HB	0.60
Harth	2008	Germany	Caucasian	PCR-melting-curve	145	122	45	HNSCC	130	138	32	HB	0.62
Jain	2006	India	Asian	PCR-RFLP	46	23	7	ESCC	72	56	9	HB	0.67
Jourenkova	1999a	France	Caucasian	PCR-RFLP	49	53	15	HNSCC	86	64	22	HB	0.07
Jourenkova	1999b	France	Caucasian	PCR-RFLP	62	52	15	HNSCC	86	64	22	HB	0.07
Jourenkova	1998	France	Caucasian	PCR-RFLP	46	41	11	LSCC	86	64	22	HB	0.07
Kelders	2002	Netherlands	Caucasian	PCR-RFLP	36	38	13	HNSCC	26	18	7	HB	0.20
Kihara	1999	Japan	Asian	PCR-RFLP	84	32	9	LSCC	184	65	8	HB	0.45
Larsen	2006	Australia	Caucasian	PCR-RFLP	230	213	51	LSCC	161	169	49^a^	HB	0.66
		Australia	Caucasian	PCR-RFLP	230	213	51	LSCC	112	100	35^b^	PB	0.11
Leichsenring	2006	Brazil	Mixed	PCR-RFLP	30	34	8	HNSCC	30	25	5	PB	0.95
Leite	2007	Brazil	Mixed	PCR-RFLP	14	13	2	SSCC	60	46	18	PB	0.07
Lewis	2002	UK	Caucasian	PCR-RFLP	14	17	1	LSCC	64	74	13	HB	0.19
Li	2010	South African	Black African	PCR-RFLP	56	59	26	ESCC	76	83	27	PB	0.58
			Mixed	PCR-RFLP	34	52	11	ESCC	30	51	13	PB	0.24
Li	2007	USA	Caucasian	PCR-RFLP	336	356	111	HNSCC	333	385	121	PB	0.57
Liang	2005	China	Asian	diASA-AMP	58	32	4	LSCC	132	86	9	HB	0.27
Liu	2010	China	Asian	PCR-RFLP	66	29	0	ESCC	61	27	3	PB	1.00
Malik	2010	India	Asian	PCR-RFLP	53	36	14	ESCC	111	75	9	PB	0.41
Matejcic	2011	South African	Black African	TaqMan genotyping	79	155	91	ESCC	100	242	132	PB	0.57
		South African	Mixed	TaqMan genotyping	69	112	48	ESCC	145	191	92	PB	0.05
McWilliams	2000	USA	Mixed	PCR-RFLP	60	73	13	HNSCC	58	51	15	HB	0.47
Miller	2006	USA	Caucasian	PCR-RFLP	190	173	49	LSCC	579	623	141	PB	0.16
Moaven	2010	Iran	Asian	PCR-RFLP	84	50	14	ESCC	74	54	8	PB	0.65
Nazar	2003	USA	Mixed	PCR-RFLP	35	29	9	LSCC	199	234	54	PB	0.23
Olshan	2000	USA	Mixed	PCR-RFLP	40	62	7	HNSCC	68	80	20	HB^c^	0.63
		USA	Mixed	PCR-RFLP	18	38	7	HNSCC	7	13	5	HB^d^	0.82
Oude	2003	Netherlands	Caucasian	PCR-RFLP	116	90	29	HNSCC	125	121	39	PB	0.27
Peters	2006	USA	Mixed	PCR-RFLP	303	311	76	HNSCC	333	329	86	PB	0.73
Ramsay	2001	UK	Caucasian	SSCP	10	10	0	SSCC	53	71	17	HB	0.36
Risch	2001	Germany	Caucasian	PCR-RFLP	76	77	18	LSCC	167	151	35	HB	0.92
Rossini	2007	Brazil	Mixed	PCR-RFLP	42	65	18	ESCC	116	108	28	PB	0.71
Ruwali	2009	India	Caucasian	PCR-RFLP	224	112	14	HNSCC	199	138	13	PB	0.06
Ruwali	2011	India	Caucasian	PCR-RFLP	316	162	22	HNSCC	285	195	20	PB	0.06
Ryberg	1997	Norway	Caucasian	PCR-RFLP	20	34	13	LSCC	153	117	27	PB	0.50
Schneider	2004	Germany	Caucasian	PCR-melting-curve	81	75	27	LSCC	298	254	70	PB/HB	0.16
Soucek	2010	Czech/Polish	Caucasian	TaqMan drug metabolism genotyping	56	53	7	HNSCC	57	50	10	PB	0.52
Soya	2007	India	Asian	PCR-RFLP	219	162	27	*UADTSCC*	120	88	12	PB	0.42
Stücker	2002	France	Caucasian	PCR-RFLP	54	46	15	LSCC	124	120	20	HB	0.22
Tan	2000	China	Asian	PCR-RFLP	93	48	9	ESCC	91	53	6	PB	0.62
To	2002	Spain	Caucasian	PCR-RFLP	101	84	19	HNSCC	100	78	23	PB	0.20
To	1999	Spain	Caucasian	PCR-RFLP	29	20	3	LSCC	64	54	14	PB^b^	0.61
		Spain	Caucasian	PCR-RFLP	29	20	3	LSCC	90	90	20	PB^e^	0.72
van	1999	Netherlands	Caucasian	PCR-RFLP	5	6	2	ESCC	146	89	12	PB	0.74
Zendehdel	2009	Sweden	Caucasian	Pyrosequencing	26	42	10	ESCC	208	207	38	PB	0.18

*PCR* polymerase chain reaction, *PCR-RFLP* polymerase chain reaction-restriction fragment length polymorphism, *diASA-AMP* di-allele-specific-amplification with artificially modified primers assay, *SSCP* Single-stranded conformational polymorphism, *ESCC* oesophageal squamous cell carcinoma, *HNSCC* head and neck squamous cell carcinoma, *LSCC* lung squamous cell carcinoma, *SSCC* skin squamous cell carcinoma, *OSCC* oral squamous cell carcinoma, *UADTSCC* upper aerodigestive tract squamous cell carcinoma, *PB* population-based, *HB* hospital-based, *P*_HWE_ *P* value of hardy-weinberg equilibrium

^a^COPD patients without LSCC, ^b^healthy smokers; ^c^control from Caucasian population; ^d^control from Black African population; ^e^control from general population

### Overall meta-analysis

First, we performed the overall meta-analysis, which included 52 case-control studies with 9763 cases and 15,028 controls (Table 2). The fixed-effects model was applied in all meta-analyses, because no substantial between-study heterogeneity was detected [Table 2, I^2^ value < 50.0%, *P*_h_ > 0.1]. As shown in Table 2, no altered susceptibility to SCC disease in cases was observed compared with controls [allele, *P*_a_ = 0.601; carrier, *P*_a_ = 0.587; homozygote, *P*_a_ = 0.689; heterozygote, *P*_a_ = 0.167; dominant, *P*_a_ = 0.289; dominant, *P*_a_ = 0.548]. These data suggest that the rs1695 polymorphism within the *GSTP1* gene does not contribute to the risk of overall SCC.

**Table 2:** Meta-analysis of the *GSTP1* rs1695 A/G polymorphism

Statistical analysis	Index	Allele	Carrier	Homozygote	Heterozygote	Dominant	Recessive
Association	OR	0.99	0.99	1.02	0.96	0.97	1.03
	95% CIs	0.95~1.03	0.94~1.03	0.93~1.12	0.91~1.02	0.92~1.03	0.94~1.12
	*P*_a_	0.601	0.587	0.689	0.167	0.289	0.548
Sample size	case	9763	9763	9763	9763	9763	9763
	control	15,028	15,028	15,028	15,028	15,028	15,028
	study	52	52	52	52	52	52
Heterogeneity	I^2^	15.5%	0.0%	9.7%	7.7%	11.8%	1.2%
	*P*_*h*_	0.174	0.999	0.278	0.318	0.239	0.450
	Model	Fixed	Fixed	Fixed	Fixed	Fixed	Fixed
Egger’s test	t	1.14	1.38	0.13	2.36	2.16	−0.31
	*P*_*E*_	0.259	0.175	0.899	0.022	0.036	0.760
Begg’s test	z	0.53	0.84	0.77	1.96	1.82	1.29
	*P*_*B*_	0.597	0.398	0.444	0.049	0.068	0.198

*OR* odds ratio, *CIs* confidence intervals, *P*_a_, *P* value of association test, *P*_h_, *P* value of heterogeneity test, *P*_E_, *P* value of Egger’s test, *P*_B_, *P* value of Begg’s test

### Subgroup analysis

**Fig. 2:**
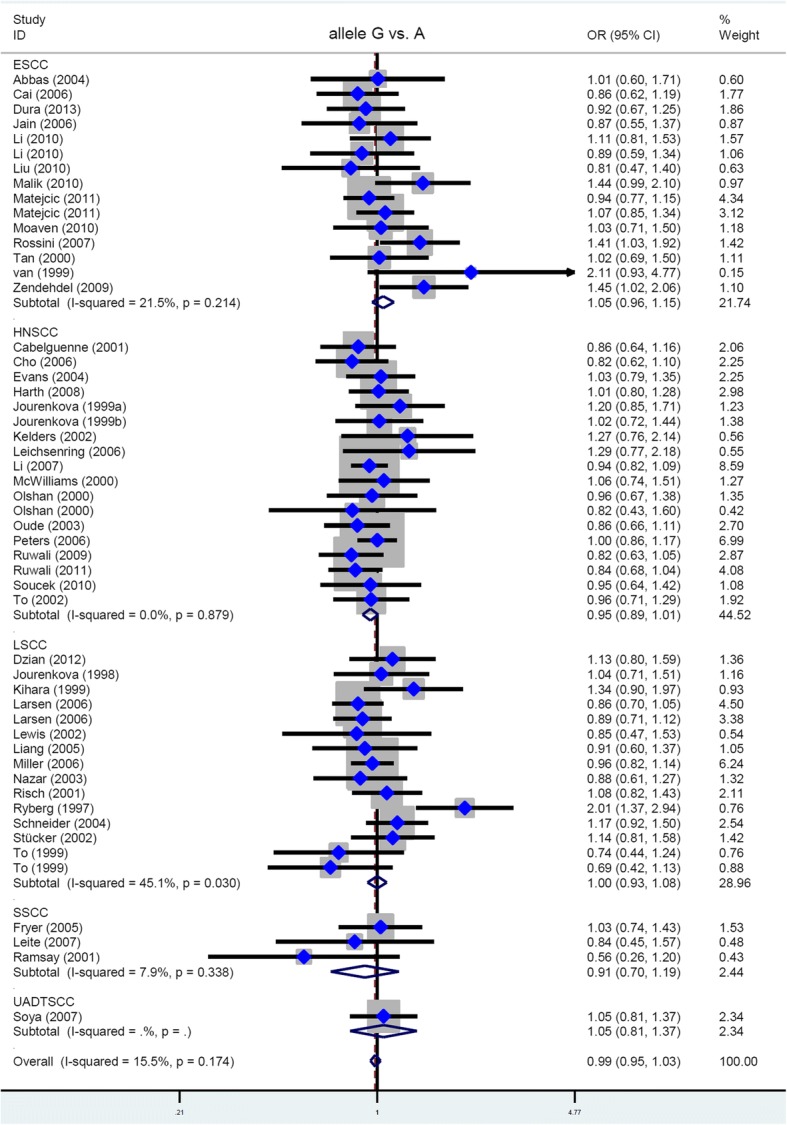
Data of subgroup analysis by SCC type (allele model)

Next, we performed additional subgroup meta-analyses according to the factors of race (Asian/Caucasian), genotyping assay (PCR-RFLP), control source (PB/HB), and disease type (ESCC/HNSCC/LSCC/SSCC). As shown in Tables 3 and 4, there were no significant associations in any subgroup analysis for all genetic models tested (all *P*_a_ > 0.05). The forest plot of the subgroup analysis by disease type under the allele model is shown in Fig. 2.

**Table 3:** Subgroup analysis of the *GSTP1* rs1695 A/G polymorphism by race, genotyping assay and control source

Factor	Subgroup	Index	Allele	Carrier	Homozygote	Heterozygote	Dominant	Recessive
Race	Asian	OR (95% CIs)	1.00 (0.89~1.12)	0.98 (0.86~1.11)	1.29 (0.94~1.76)	0.90 (0.78~1.04)	0.94 (0.82~1.08)	1.35 (0.99~1.83)
		*P*_a_	0.948	0.716	0.114	0.139	0.361	0.058
		Case/control	1696/2139	1696/2139	1696/2139	1696/2139	1696/2139	1696/2139
		Study number	10	10	10	10	10	10
Race	Caucasian	OR (95% CIs)	0.98 (0.93~1.03)	0.98 (0.82~1.04)	1.00 (0.89~1.12)	0.94 (0.87~1.01)	0.95 (0.89~1.02)	1.02 (0.91~1.14)
		*P*_a_	0.358	0.447	0.984	0.099	0.153	0.716
		Case/control	5968/9719	5968/9719	5968/9719	5968/9719	5968/9719	5968/9719
		Study number	30	30	30	30	30	30
genotyping assay	PCR-RFLP	OR (95% CIs)	0.99 (0.94~1.03)	0.99 (0.93~1.04)	1.01 (0.91~1.12)	0.96 (0.90~1.03)	0.97 (0.91~1.03)	1.01 (0.91~1.12)
		*P*_a_	0.542	0.579	0.874	0.260	0.351	0.824
		Case/control	8008/11,342	8008/11,342	8008/11,342	8008/11,342	8008/11,342	8008/11,342
		Study number	42	42	42	42	42	42
control source	PB	OR (95% CIs)	0.98 (0.94~1.03)	0.98 (0.93~1.04)	1.00 (0.90~1.12)	0.96 (0.89~1.03)	0.96 (0.90~1.03)	1.02 (0.92~1.13)
		*P*_a_	0.519	0.572	0.943	0.214	0.287	0.751
		Case/control	6697/10,170	6697/10,170	6697/10,170	6697/10,170	6697/10,170	6697/10,170
		Study number	31	31	31	31	31	31
control source	HB	OR (95% CIs)	0.98 (0.91~1.06)	0.98 (0.90~1.07)	1.00 (0.84~1.20)	0.95 (0.86~1.06)	0.96 (0.87~1.07)	1.01 (0.85~1.19)
		*P*_a_	0.586	0.638	0.977	0.377	0.461	0.944
		Case/control	2771/3946	2771/3946	2771/3946	2771/3946	2771/3946	2771/3946
		Study number	19	19	19	19	19	19

*P*_a_, *P* value of association test

*PCR-RFLP* polymerase chain reaction-restriction fragment length polymorphism, *PB* population-based, *HB* hospital-based, *OR* odds ratio, *CIs* confidence intervals

**Table 4:** Subgroup analysis of the *GSTP1* rs1695 A/G polymorphism by SCC type

Subgroup	Index	Allele	Carrier	Homozygote	Heterozygote	Dominant	Recessive
ESCC	OR (95% CIs)	1.05 (0.96~1.15)	1.03 (0.93~1.14)	1.15 (0.95~1.39)	1.00 (0.88~1.14)	1.03 (0.92~1.17)	1.13 (0.95~1.34)
	*P*_a_	0.263	0.568	0.155	0.970	0.575	0.160
	Case/control	1934/3951	1934/3951	1934/3951	1934/3951	1934/3951	1934/3951
	Study number	15	15	15	15	15	15
HNSCC	OR (95% CIs)	0.95 (0.89~1.01)	0.96 (0.89~1.03)	0.94 (0.82~1.09)	0.94 (0.87~1.02)	0.93 (0.86~1.01)	0.95 (0.83~1.09)
	*P*_a_	0.112	0.247	0.408	0.131	0.102	0.459
	Case/control	4671/4961	4671/4961	4671/4961	4671/4961	4671/4961	4671/4961
	Study number	18	18	18	18	18	18
LSCC	OR (95% CIs)	1.00 (0.93~1.08)	1.00 (0.92~1.09)	1.04 (0.88~1.24)	0.97 (0.87~1.07)	0.98 (0.89~1.09)	1.06 (0.90~1.25)
	*P*_a_	0.940	0.973	0.616	0.526	0.741	0.485
	Case/control	2574/5421	2574/5421	2574/5421	2574/5421	2574/5421	2574/5421
	Study number	15	15	15	15	15	15
SSCC	OR (95% CIs)	0.91 (0.70~1.19)	0.94 (0.69~1.28)	0.83 (0.46~1.49)	0.94 (0.64~1.36)	0.91 (0.64~1.30)	0.86 (0.49~1.51)
	*P*_a_	0.493	0.688	0.532	0.728	0.605	0.597
	Case/control	177/475	177/475	177/475	177/475	177/475	177/475
	Study number	3	3	3	3	3	3

*ESCC* oesophageal squamous cell carcinoma, *HNSCC* head and neck squamous cell carcinoma, *LSCC* lung squamous cell carcinoma, *SSCC* skin squamous cell carcinoma, *OR* odds ratio, *CIs* confidence intervals, *P*_a_, *P* value of association test

Furthermore, we included all case-controls studies regarding the specific SCC type and conducted a series of subgroup analyses by race and control source. However, similar results were obtained (data not shown). As a result, the *GSTP1* gene rs1695 polymorphism is not likely related to the genetic susceptibility of a specific SCC type, including ESCC, HNSCC, LSCC, and SSCC.

### Publication bias and sensitivity analysis

**Fig. 3:**
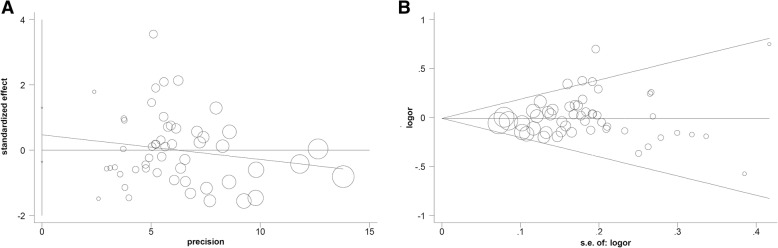
Funnel plot of publication bias analysis. **a** Egger’s test; **b** Begg’s test

**Fig. 4:**
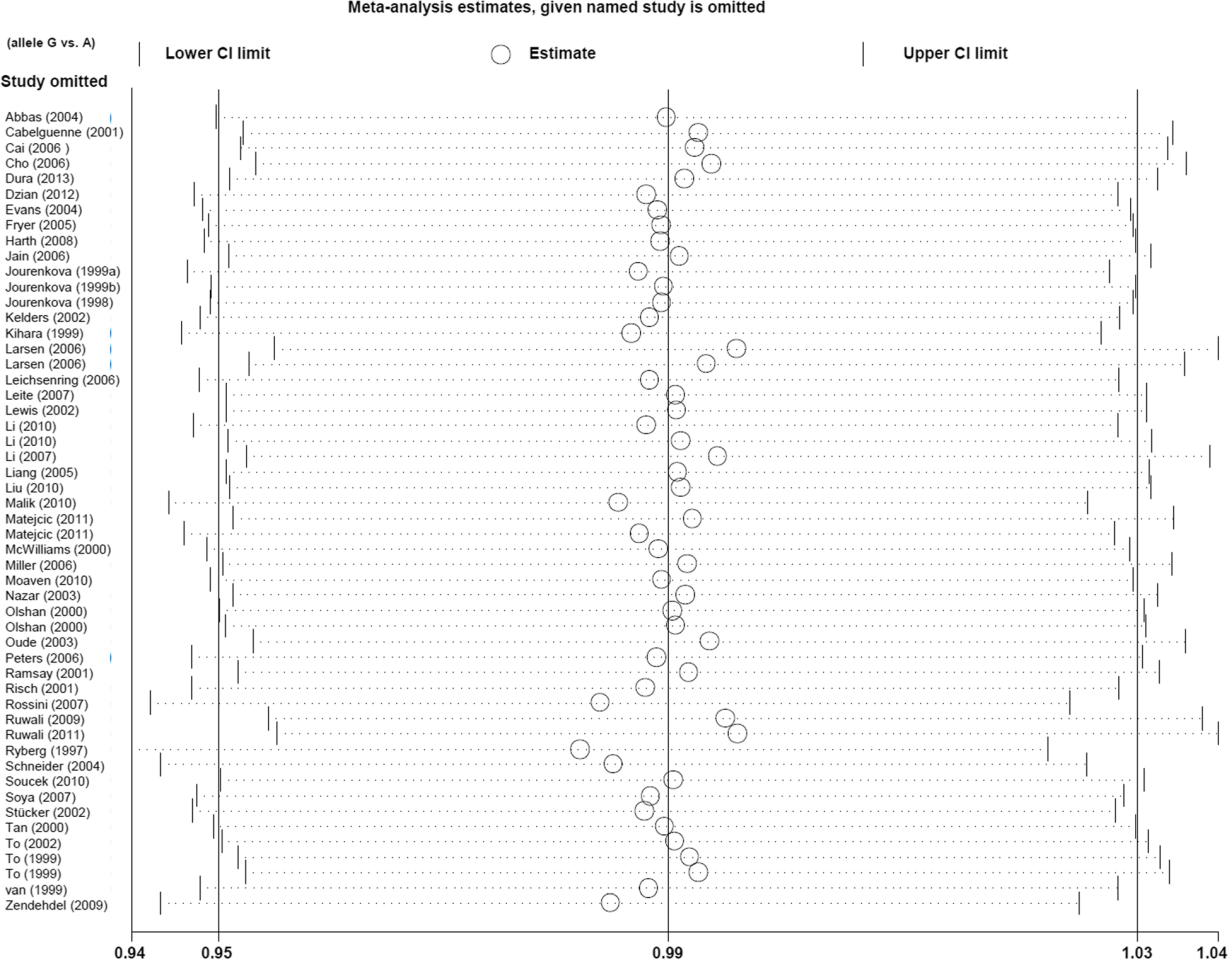
Sensitivity analysis data (allele model)

The publication bias analysis data obtained from Egger’s and Begg’s tests are shown in Table 2. There was no remarkable publication bias in most genetic models (*P*_E_ > 0.05, *P*_B_ > 0.05), except for the heterozygote (*P*_E_ = 0.022, *P*_B_ = 0.049) and dominant (*P*_E_ = 0.036) models. The funnel plot (allele model) is displayed in Fig. 3a-b. Moreover, our sensitivity analysis led us to consider the stability of the data. Figure 4 shows a representative example of the sensitivity analysis (allele model).

## Discussion

In the current meta-analysis, we first focused on the genetic relationship between the *GSTP1* rs1695 A/G polymorphism and the risk of overall SCC and then conducted subgroup analyses by the specific histological status. After rigorous screening, four main types of SCC, namely, ESCC, HNSCC, ESCC, and SSCC, were targeted.

ESCC, a type of squamous epithelium differentiation of a malignant tumour within the oesophagus, accounts for the vast majority of oesophageal cancers [64, 65]. ESCC often presents in physiological or pathological stenosis of the oesophagus, and genetic factors, carcinogens, and/or chronic irritants may contribute to the pathogenesis of ESCC [64, 65]. The *GSTP1* rs1695 A/G polymorphism is significantly related to the risk of ESCC in the Kashmiri population [42]. Similarly, *GSTP1* rs1695 may be an independent risk factor for ESCC in Western populations [53]. Nevertheless, different associations were detected in other reports. For instance, no difference between unrelated controls and ESCC cases was observed in a French population [14] or a Chinese population [61]. Therefore, a meta-analysis was required to comprehensively evaluate the role of the *GSTP1* rs1695 A/G polymorphism in ESCC risk. Herein, we recruited 15 case-control studies involving 1934 cases and 3951 controls and performed a new meta-analysis to examine the association between the *GSTP1* rs1695 A/G polymorphism and ESCC susceptibility. The carrier (carrier G vs. A) model, as well as the allele, homozygote, heterozygote, dominant and recessive genetic models, was used. Our results in the stratified analysis of specific ESCCs are consistent with the data of Tan et al. [18].

Similarly, inconsistent results regarding an association between the *GSTP1* rs1695 A/G polymorphism and LSCC risk have been reported in different races and geographical locations [24, 31, 33, 34, 37, 40, 45, 47, 52, 56, 57, 60, 63]. Here, we failed to detect a positive correlation between *GSTP1* rs1695 and LSCC susceptibility, consistent with the prior meta-analysis of Feng in 2013 [66] and Xu in 2014 [67].

Head and neck cancer comprises cancers of the mouth, nose, sinuses, salivary glands, throat, and lymph nodes in the neck, and HNSCC is the major pathologic type [68]. In 2012, Lang et al. enrolled 28 case-control studies to perform a meta-analysis regarding the genetic effect of the *GSTP1* rs1695 A/G polymorphism on overall head and neck cancer [69]. The authors were unable to identify a positive association between the *GSTP1* rs1695 A/G polymorphism and the risk of overall head and neck cancer. Nevertheless, the potential role of *GSTP1* rs1695 in the susceptibility to HNSCC was not assessed. Therefore, we performed a subgroup meta-analysis of HNSCC involving 18 case-control studies, but did not identify an association between *GSTP1* rs1695 and HNSCC risk.

SSCC, SBCC (skin basal cell carcinoma) and (MM malignant melanoma) are the three main types of cutaneous cancer [4]. Herein, we did not identify an association between the *GSTP1* rs1695 A/G polymorphism and SSCC risk, consistent with the prior meta-analyses regarding the correlation between *GSTP1* rs1695 and the susceptibility to cutaneous cancer in 2015 [70, 71].

Human GST family genes, mainly including *GSTA* (glutathione S-transferase alpha), *GSTM1* (glutathione S-transferase mu 1), *GSTT1* (glutathione S-transferase theta 1) and *GSTP1*, encode phase II enzymes and are thus important for the body defence, metabolic detoxification of mutagens or chemical drugs, or cellular elimination of carcinogens [9, 10]. The rs1695 A/G polymorphism within the *GSTP1* gene can result in the substitution of Ile (isoleucine) for Val (valine) at amino acid position 105, which may lower the cytosolic enzyme activity of *GSTP1* protein [72, 73]. Although significant associations were not obtained in our overall meta-analysis or subgroup analyses by pathological type, we cannot rule out the potential genetic effect of the *GSTP1* rs1695 A/G polymorphism.

There are still some limitations to our meta-analysis that should be clarified. Even though our findings were considered reliable by our sensitivity analysis and publication bias assessment, more eligible investigations are still warranted to further enhance the statistical power. We note that population-based controls were not utilized in each case-control study. The currently available data of genotypic and allelic frequency from the on-line databases led us to only target the rs1695 polymorphism of the *GSTP1* gene. Other possible functional polymorphisms of the *GSTP1* gene, such as rs1138272, or relative haplotypes will be important to examine in the future. We should also pay attention to the genetic relationship between GSTP1/GSTM1/GSTT1 polymorphisms and the risk of SCC.

## Conclusion

In general, based on the currently published data, the *GSTP1* gene rs1695 polymorphism is not associated with the susceptibility to overall SCC diseases, including ESCC, HNSCC, LSCC, and skin SCC. The confirmation or refutation of this conclusion merits further evidence.

## Abbreviations

AHR: Aryl hydrocarbon receptor
CADM1: Cell adhesion molecule 1
diASA-AMP: Di-allele-specific- amplification with artificially modified primers assay
Embase: Excerpta Medica Database
ESCC: Oesophageal squamous cell carcinoma
GST: Glutathione S-transferase
GSTA: Glutathione S-transferase alpha
GSTM1: Glutathione S-transferase mu 1
GSTP1: Glutathione S-transferase pi 1
GSTT1: Glutathione S-transferase theta 1
GWAS: Genome-wide association study
HB: Hospital-based
HNSCC: Head and neck squamous cell carcinoma
HWE: Hardy-Weinberg equilibrium
KLF5: Kruppel like factor 5
LSCC: Lung squamous cell carcinoma
MM: Malignant melanoma
OSCC: Oral squamous cell carcinoma
PB: Population-based
PCR: Polymerase chain reaction
PCR-RFLP: Polymerase chain reaction-restriction fragment length polymorphism
SBCC: Skin basal cell carcinoma
SCC: Squamous cell carcinoma
SEC16A: SEC16 homolog A, endoplasmic reticulum export factor
SSCC: Skin squamous cell carcinoma
SSCP: Single-stranded conformational polymorphism
UADTSCC: Upper aerodigestive tract squamous cell carcinoma
WOS: Web of Science
